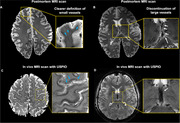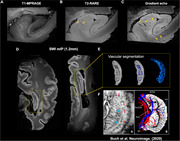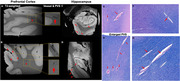# Intrinsic Contrast from Blood Clots in Postmortem Vascular MRI

**DOI:** 10.1002/alz70855_102281

**Published:** 2025-12-23

**Authors:** Chenyang Li, Huize Pang, Annie Li, Dominique Leitner, Thomas Wisniewski, Mary Bruno, Youssef Zaim‐Wadghiri, Jiangyang Zhang, Yulin Ge

**Affiliations:** ^1^ NYU Grossman School of Medicine, New York, NY, USA; ^2^ New York University Grossman School of Medicine, New York, NY, USA; ^3^ Center for Cognitive Neurology, New York University Langone Health, New York, NY, USA; ^4^ Alzheimer's Disease Research Center, New York University Langone Health, New York, NY, USA

## Abstract

**Background:**

To interpret clinical Alzheimer's disease (AD) pathology, postmortem MRI is frequently used to provide crucial insights into brain structure and vasculature, bridging *in vivo* imaging and histopathological validation. However, imaging blood and vascular changes after death and fixation remains poorly understood. Traditional vascular contrast agents are ineffective in postmortem imaging studies due to lack of circulation, posing challenges for vascular mapping of postmortem tissues. This study is to explore how blood clots can be used as contrast media to enhance postmortem vascular MRI.

**Method:**

We studied three human brain tissues with postmortem diagnosis of epilepsy, Parkinson's Disease and AD, acquired from the NYU ADRC. The brain hemisphere was scanned first using human whole‐body 7T scanner. This is followed by cutting the hemisphere into smaller tissue chunks, including the prefrontal cortex and hippocampus, for high resolution preclinical MRI on animal scanners. Subsequently, histopathological staining, using hematoxylin and eosin (H&E) and Luxol fast blue stains (LFB), were performed to evaluate the vascular morphology and blood clot formation.

**Result:**

High resolution susceptibility weighted imaging (SWI) of post‐mortem brain revealed clear small vessel contrast in grey matter and white matter, comparable to in vivo USPIO‐enhanced 7T MRI (Figure 1A and C). Moreover, intrahippocampal vasculatures were well illustrated in postmortem MRI, closely resembling USPIO‐enhanced clinical MRI findings (Figure 2E‐FC). Histopathology staining (HE and LFB staining) of hippocampal tissue demonstrated the blood clot vascular structures in the white matter, hippocampus and cortical regions (red arrow in Figure 3).

**Conclusion:**

This study demonstrates the use of postmortem coagulated blood as an intrinsic contrast agent on T2*‐weighted images for visualizing small vasculatures, establishing a method for detailed vascular mapping in postmortem specimens alongside histopathological analysis. This approach can help to enhance our understanding of *in vivo* vascular imaging findings of vascular contributions to AD and AD‐related dementia and their underlying pathological substrates.

**Figure 1**. Comparison of high‐resolution T2*‐weighted data between postmortem and *in vivo MRI*.

**Figure 2**. Multi‐contrast MRI of post‐mortem hippocampus compared with histopathology and in vivo vascular MRI.

**Figure 3**. Blood clot formation (arrows) in postmortem brain tissue on T2*‐weighted imaging and histological staining.